# How the Health Rumor Misleads People’s Perception in a Public Health Emergency: Lessons from a Purchase Craze during the COVID-19 Outbreak in China

**DOI:** 10.3390/ijerph17197213

**Published:** 2020-10-02

**Authors:** Liwei Zhang, Kelin Chen, He Jiang, Ji Zhao

**Affiliations:** 1 School of Public Administration, Jilin University, Changchun 130012, China; zhanglw@jlu.edu.cn; 2 Institute of Urban Governance, Shenzhen University, Shenzhen 518060, China; chenkelin@szu.edu.cn; 3 Department of Social Psychology, Nankai University, Tianjin 300350, China; 1120190824@mail.nankai.edu.cn; 4 School of International and Public Affairs, Shanghai Jiao Tong University, Shanghai 200030, China

**Keywords:** health rumor, health perception, health communication, public health emergency, risk governance, COVID-19

## Abstract

Health rumors often mislead people and cause adverse health behaviors. Especially during a public health emergency, health rumors may result in severe consequences for people’s health and risk governance. Insight into how these rumors form and harm people’s health behavior is critical for assisting people in establishing scientific health cognition and to enhance public health emergency responses. Using the case study with interview data of a salient purchase craze led by a health rumor during the COVID-19 outbreak in China, this article aimed to illustrate the process of how a piece of information becomes a health rumor. Furthermore, we identify factors that cause people to believe rumors and conduct behavior that leads to a purchase craze. Results show that a public misunderstanding of the unique psychology of uncertainty, cultural and social cognition, and conformity behavior jointly informs people’s beliefs in rumors and further causes purchase craze behavior. We developed a simplified model to demonstrate how an ordinary news report can lead to a rumor. Based on this model, some implications of effective health communication are suggested for managing rumors.

## 1. Introduction

In the public health field, the public is prone to learning about health and investigating their own health conditions by obtaining massive amounts of health information. However, if many pieces of such widespread information are inaccurate or even totally wrong, people can easily become misinformed and conduct wrongful health behaviors [1,2]. In this article, such inaccurate or incorrect information is defined as a rumor, which always thrive during crises. From the SARS (severe acute respiratory syndrome) outbreak to the Ebola outbreak and further to the current COVID-19 outbreak, various rumors can quickly spread through a range of media and communication channels that influence people’s risk perception and mislead people’s behaviors [3,4,5,6,7]. During the COVID-19 outbreak, viral dissemination of rumors not only hurt people’s perceptions but also wrecked risk management. As the World Health Organization (WHO) put forward, the world is combating both the spread of the disease and an “infodemic” [8].

Academics have reached a consensus that the study of rumors associated with public health contributes to the sociology, management, and communication science of health by enhancing our understanding of lay health knowledge and beliefs, as well as the situated basis of health-relevant actions. Academics also recommend some communication methods for tackling and correcting health rumors [4,9,10,11,12,13,14]. However, in order to keep people from believing these rumors, it is crucial to apply effective health communication to eliminate these rumors during a public health emergency as ex ante prevention instead of as an ex post response. Therefore, it is necessary to understand how health rumors form and spread during a public health emergency, which enables us to suggest recommendations of health communication.

This article mainly analyses the formation of health rumor and how the rumor impacts people’s perception and behavior in a public health emergency. We utilized a case with interview data from interviewees (N = 30) that focuses on a salient purchase craze of Shuanghuanglian (SHL), a traditional Chinese medicine (TCM), led by a health rumor during the COVID-19 outbreak in China. The reason why we selected China’s case is dependent on that COVID-19 outbreak firstly occurred in China, which means China had severely insufficient knowledge about the novel coronavirus in this specific period. In such situation, the public could be misled by health rumor easily [4,15]. Therefore, it is appropriate to observe and analyze the formation mechanism and impact of the rumor. Interestingly, according to a statistic of COVID-19 rumors, in addition to SHL, many medicines are asserted to have positive effects on preventing or curing COVID-19, such as Ivermectin, Remdesivir, Aspirin, etc. However, in China, only SHL stimulated people’s purchase craze and no report or phenomenon showed people’s strong desire of buying other medicines mentioned by rumors. Thus, why the Chinese people prefer SHL should be discussed.

In order to mention effective health communication for preventing and managing rumors, this article also analyzes the formation mechanism of the rumor. This article is dedicated to offering a holistic process involving the formation, dissemination, and management of rumors during a public health emergency.

## 2. Literature Review

### 2.1. Conceptualizing Rumor

The rumor has become an enduring feature of social and organizational landscapes [16]. As a concept, rumor has synonyms that represent inaccurate or false information, such as misinformation and disinformation. In order to avoid confusion of these concepts, it is critical to clarify them and define the concept of a rumor in this article.

In the public health domain, commonly used concepts concerning inaccurate or false information are “misinformation” and “disinformation”. Generally, health misinformation is contrary to the epistemic consensus of the scientific community regarding a phenomenon. However, what is considered true and false is constantly changing as new evidence comes to light and as techniques and methods are advanced [1,17]. Therefore, scholars argue that such definitions are seen as a relatively narrow slice of untrue information. An inclusive connotation should include unverified, vague, or contradictory information, and the nonfactual claim being made—and misinformation beliefs, or misperceptions—that misinformation claims are true [18,19]. As for disinformation, disinformation is regarded as intentionally false or inaccurate information that is spread deliberately with misinformation, which is unintentionally false information [1,20,21,22]. The intention to create disinformation usually stems from earning money, public attention, as well as clicks or followers, especially in social media environments [1,20].

An example of existing classical research specifically focusing on rumors is Allport and Postman’s paper. They indicated that the rumor thrives on a lack of news. It is a proposition or belief, passed along from person-to-person, usually by word-of-mouth, without secure standards of evidence [23,24]. Admittedly, developed communication media in modern society has formed more vectors of rumor spreading, such as social media [25]. Other literature follows such a definition and regards rumors as unverified and instrumentally relevant information statements in circulation that arise in contexts of ambiguity, danger, or potential threat that function to help people make sense of and manage risk [16].

The controversial discussion around rumors include the containment relation of rumor and misinformation, even though some scholars use them as interchangeable concepts [26]. The relevant literature indicates that rumor is a form of misinformation, just like hoaxes and conspiracy theories. However, some scholars used Procter and Gamble’s case to argue that rumor includes so-called misinformation or false statements thought to be true by some people. The implications of this reflection are sobering and lead to a general sense of caution about what is heard; evidence that initially seems firm may in fact easily crumble [16,27].

Therefore, we tend to use the concept of rumors to illustrate a process of information distortion in transmission [28,29]. Allport and Postman argued that the expression, content, and structure of a rumor may be changed when traveling from person-to-person [30]. Likewise, a piece of information with an accurate original meaning may be distorted as well. For instance, an individual’s orientation to a rumor draws upon both personal resources to evaluate rumors as well as social beliefs and personal attitudes about reproduction that influence the initial disposition toward the rumor [31]. To sum up, the information-receiver may play the role of rumor-former when the receiver retransmits the information, because the retransmission version may incorporate personal cognition and perception. Any misperception results in information distortion, which enhances a rumor’s uncertainty and inaccuracy.

### 2.2. People’s Perception and Health Behavior

It is common knowledge that a specific perception will lead to specific health behavior. However, the factors that impact the perception and behavior are complex [32,33]. There are strong theoretical and empirical studies for illustrating what sorts of factors may influence people’s perception and motivate them to make health-related decisions.

For an individual, health literacy is the main factor for shaping personal health perception. Health literacy focuses on increasing responsibility of individuals and groups to be able to understand and act on the health information. It represents an ability to identify and use health information and to make the decision for improving healthy life [34,35,36]. For example, a person with a good health literacy can read and understand health-related texts, to find and interpret information in the documents, such as to conduct correct and scientific behavior to control health risk and change health status. Generally, health literacy not only comprises writing or reading health-related things but also their implementation [36,37].

In the perspective of rational choice economics, people’s perceptions and related behavior are dependent on expected value, which means a person will identify and calculate all possible positive and negative outcomes of the decision/behavior, and select a best one amongst all potential outcomes [33,38]. Scholars also argue different conditions that influence people’s perceptions and judgments. For instances, people’s perceptions and judgments can be influenced by prior information and, when provided with new information [33,39,40]. In addition, people judge events as more frequent when those events are more easily imagined and brought to mind [41].

Culture, broadly defined as what is learned, shared, transmitted intergenerationally, and reflected in a group’s values, beliefs, norms, behaviors, communication, and social roles, can affect health-related behaviors both directly and indirectly as well [42,43]. For example, TCM profoundly impacts Chinese people’s health belief and behavior, and local religions in some countries also lead to people’s special health behaviors [44,45].

Health communication is critical to helping people reduce their health risks, as well as to receiving more significant benefits in return for risks they take. It can ensure that information receivers establish an accurate perception and conduct rational behavior. When resorting to risk communications, messages should be shared openly and timely, aiming to bridge the knowledge gap between the originators of information and those receiving the information, and should adjust the public’s behavior to cope with the risk [46,47,48].

In this paper, we will bridge the health rumor, personal perception, and health behavior, to illustrate how a rumor misleads people’s perception and further impacts health behavior. We also discuss strategies of effective health communication for forming or correcting people’s perceptions. Focusing on the specific COVID-19 era, we aimed to offer the implication of health communication for combating the “infodemic” during this COVID-19 outbreak. From China’s context, we can also realize the specialty in health behavior, which enables us to broaden our knowledge about the character of health perception and behavior in the cross-cultural or cross-national situation.

## 3. Method and Materials

### 3.1. Background and Media Resources

The internet has become a primary channel through which people obtain health information. Especially considering social media, it is becoming a reliable platform for health communication to target audiences due to its promptness, multi-sources and diverse contents [49]. In China, social media is also a credible source of health information for the public, and during the COVID-19 era, the Chinese people have used the social media to receive updates of the outbreak [50]. The case in this article is related to a rumor in the social media environment, which further misled people’s perception and behavior. In this article, we utilized media resources to describe this entire purchase craze event. The process-tracing of this purchase craze was mainly summarized by reports from China’s authoritative media outlets, such as *People News*, *China Central Television*, *China News*, *Sohu News*, *Sina News* and *Tencent News*. Some dissemination contents related to SHL came from Chinese social media outlets, including Weibo and WeChat.

In detail, the case was derived from a salient phenomenon during the COVID-19 outbreak in China, which was people’s purchase craze towards SHL, a TCM against influenza with symptoms such as fever, coughing, and a sore throat. At midnight on 31 January 2020, eight days after the 23 January 2000, which is the date of Wuhan’s lockdown, a social media affiliated with Chinese central official media, namely Xinhua Shidian, reported that SHL had an inhibiting effect on the novel coronavirus. Because this information came from the central official media, it was then massively reposted or transmitted by other central and local official social media and the public’s social media outlets [51]. This information promptly became a hot topic. Up to the morning of 1 February 2020, the topic “SHL can inhibit the novel coronavirus” had received approximately 440 million readings on Chinese common-used social media, namely Sina Weibo, and 180 thousand messages [52]. Such large-scale dissemination caused an irrational purchase craze across China. According to a media report, SHL in all online sales platforms was sold out within one hour, as was the poultry-based SHL [53]. Next, the public’s attentions turned to physical pharmacies. In many Chinese cities, numerous people went outside and gathered in front of pharmacies, even at midnight, instead of following the quarantine requirements and maintaining social distancing.

Although the authority made an emergent fact-checking and clarified the information, the public still persisted in purchasing SHL through different channels. However, this craze also led to tragedy. For instance, the media released news that a COVID-19 patient received the virus by being a part of a cluster of people waiting to purchase SHL [54]. This phenomenon has become a typical rumor case in the COVID-19 outbreak of China, and the media satirized this phenomenon as “Night at the SHL” [55].

### 3.2. Random Interview and Coding

In order to investigate why people irrationally purchased SHL on 1 February 2020, we conducted random interviews with people at pharmacies who wanted to purchase the medicine in three Chinese cities where the authors live in. In total, we validly interviewed 30 people. Table 1 shows the population demographics of interviewees.

During the interview, we mainly asked the six following questions:

♦ The media reported that SHL has inhibiting effects on the novel coronavirus. How do you understand the authentic meaning of this dissemination?
♦ Do you think that the concept of inhibition is similar to remedy?
♦ How do you understand the so-called inhibition?
♦ Many experts say that quarantining at home or keeping social distance is crucial. Do you fear the risk of being infected when you go outside to purchase SHL?
♦ Why do you believe SHL has a positive effect?
♦ Will you purchase other medicines if the media reports that another specific medicine has the same effect?

The intention of these six questions can be divided into three aspects: (1) to inspire people to talk as much as possible about what they think about COVID-19; (2) to realize how people clarify and understand medicine related to SHL research and COVID-19; and (3) to understand what factors stimulate people’s purchase craze behavior. In order to avoid a specific question framework that may mislead and restrict people’s real thoughts, based on the abovementioned basic questions, the interviews combined a semi-structured approach with open-ended conversations in which the interviewees were encouraged to speak freely and pose questions on the topics considered. The semi-structured component meant that certain key questions were asked in the same way in all interviews. The open-ended component was intended to capture each interviewee’s actual understanding associated with the outbreak and to uncover the real factors that influenced people’s behavior from interviewees’ discourse [56,57].

We used the ground theory to code interview materials. According to the three-phase coding framework suggested by Corbin and Strauss, the first step was open coding, followed by axial and selective coding [58]. To start, we used the line-by-line coding, which enabled us to obtain as many ideas as possible. The expressions in each interview that were considered to be relevant were coded. Codes that showed similar features were categorized. In the axial coding phase, the categories established in the open coding phase were refined and reorganized to specify properties and dimensions of a category [59]. In such a scheme, Strauss and Corbin mentioned three components that include conditions, interactions, and consequences of interactions. The objective of this phase is to form more complex clusters of categories and subcategories. In the selective coding phase, the core category was identified to underpin the essence of the phenomenon [58]. The core category is characterized by several properties, such as frequent appearance in the data, a considerable degree of abstraction, and extensive connections with all other categories and codes [60]. These core categories can completely explain the whole research, which offers a clear insight into understanding the complex phenomenon.

## 4. Results

In our interviews, every question was clearly answered by interviewees. Based on our analysis of the interview materials, the results showed four interrelated facets that influenced people’s belief in the rumors that led to irrational purchases: (1) layperson’s misunderstanding; (2) special psychology in risk; (3) social and cultural cognition; and (4) conformity behavior. Following interviewee’s answers, layperson’s misunderstanding and social and cultural cognition were the prominent factors, because almost all of the interviewees mentioned these two facets.

### 4.1. Layperson’s Misunderstanding Turns a News Report into a Rumor

During the SHL purchase craze, misunderstandings were an important element of forming and disseminating the rumor, which reflects people’s insufficient health literacy. Buyers were attracted by the news but misunderstood its authentic content. Thus, they thought that SHL was useful for curing COVID-19. The origin of the report’s title is “A research finding of the Chinese Academy of Sciences: SHL oral liquid can inhibit the novel coronavirus” [61]. However, the public also believed that SHL was able to prevent and treat the novel coronavirus because the public does not have knowledge of medical expressions, especially medical terms such as “inhibition”.

In total, 20 interviewees expressed that they only focused on the headlines and do not read full articles. One interviewee said, “The media told us good news. Our country is so strong and concludes an exciting finding. The pneumonia will be conquered soon” (S1A3). Another said, “Why do I buy SHL? The news said it can remedy the coronavirus. That is it” (S1A5). Even more, another interviewee said, “The official media states that SHL can inhibit the novel coronavirus. As for other contents, I do not care” (S3A3). Although seven people indicated that they indeed browsed entire articles rather than skimming headlines, they nonetheless failed to understand the complex and professional medical expressions being used in the articles. For example, one interviewee stated: “I went through this news, but I really have no idea what it means. In my opinion, it wants to convey that SHL can remedy the pneumonia” (S2A1). Another interviewee said, “I am not a scientist and I cannot understand what [the news] said. Take a joke, I know every [Chinese] character in this news, but I know nothing of how these [Chinese] characters connect into a sentence” (S2A2).

Among the 30 interviewees, all of them used “prevent” or “treatment” to express their understanding of the effect of SHL. Moreover, 23 interviewees failed to distinguish between the concepts of remedy and inhibition, and did not possess the knowledge to understand the medical expressions and terms that the news conveyed. With respect to the distinction between “inhibition” and “prevention”, for instance, some interviewees said: “Inhibition certainly means prevention” (S1A1); “I do not understand so-called inhibition, but I think SHL can prevent the coronavirus” (S1A9); “Inhibition, semantically, naturally means restriction. If you drink SHL, you can keep away from the virus” (S3A6); “I think [inhibition and prevention] are different. However, in essence, no matter what meanings they are, it is the same for curing the disease” (S2A9). Furthermore, five interviewees said that the inhibition meant that SHL could defeat the virus, but they were unable to explain why they held that belief. People were inclined to regard inhibition in its daily usage form instead of considering the medical expression. Some of them said: “Inhibition? Inhibition is defeat and SHL can kill the virus” (S1A10); “Perhaps, inhibition means that the ingredient of SHL can eliminate the virus under a specific chemical reaction, but I have no knowledge about this actually” (S2A7); “[Inhibition] may result in a cure. Sorry, I do not know” (S3A5); “I am not a scientist and I cannot tell the meaning [of inhibition]. Perhaps, it means therapy” (S1A8); and “Inhibition is just effective. SHL kills the coronavirus” (S1A3).

### 4.2. Special Psychology of the Uncertainty That Motivates People’s Behaviors

Following the framework of rumor spreading, four variables jointly shape people’s belief in a rumor: uncertainty, importance, lack of control, and anxiety. The uncertainty about issues of personal importance engenders feelings, such as a lack of control and anxiety [17].

19 interviewees mentioned their anxiety about COVID-19. For example, they said: “This pneumonia is too scary. If there is any hope, I want to have a try” (S1A5); “I am anxious when I saw the increasing number of deaths on TV. I think we ought to try anything we can get. The media said that SHL is effective and it is really a lifesaver” (S2A9); “Too many people died! This outbreak is more severe than SARS. The hard-working scientists give us hope and why not buy SHL to keep safe?” (S2A8) Among these interviewees, five of them expressed their fear repeatedly and emphasized that SHL was a “savior”.

Not all interviewees totally believed that SHL had a positive impact. A Chinese proverb says that “it is better to believe that it exists than not to believe”. Six interviewees stressed the importance of “hope”, even though some of them doubted whether SHL had real effectiveness. For example, an interviewee said: “Quarantine at home is so boring. In fact, I do not know if SHL has any effect, but it is a hope for us. If it is indeed effective, we can end this boring quarantine quickly and return to normal life” (S2A6). Another interviewee said: “I do not care about SHL and other medicines. I will try any medicine if it presents hope” (S2A10).

### 4.3. Belief in Traditional Chinese Medicine Is an Important Social and Cultural Factor

Cultural factors play a vital role in this purchase craze. The public’s belief in SHL stems from the fact that Chinese people trust the TCM. A similar story happened during the SARS outbreak in China in 2003, wherein a purchase craze of Banlangen occurred, which is another widely-used TCM [62]. A national survey in 2017 showed that 94.68% of interviewees believed in traditional Chinese medicine [63]. Thus, the cognition of TCM can be seen as a particular social and cultural factor in people’s health perceptions and behavior in China, which can be observed during the purchase craze of SHL as well.

In our interviews, 27 interviewees expressed their beliefs in TCM: “TCM is a precious legacy from our Chinese ancestors. TCM even cured my rheumatism, so I think SHL is definitely effective for fighting the coronavirus” (S1A5); “TCM is miraculous and it should be effective” (S3A6); “I have no prejudice towards Western medicine, but I still believe TCM’s effect on remedying influenza. Of course, I may buy other sorts of medicine if they have an effect as well. However, TCM is always my priority” (S3A7); “SHL’s effect is common knowledge. Although it cannot defeat the virus, it may prevent infection. After all, treatment before getting illness is a rationale of TCM” (S1A9).

We also found that experts played a role in amplifying people’s perceptions. Some leading experts of China’s official expert team emphasized the advantages of TCM, which increased people’s beliefs in their effectiveness against COVID-19 [64]. In total, 13 interviewees said that experts’ statements encouraged them to purchase SHL. Some interviewee said: “Our academician told us that TCM is useful; why would we not believe these authorities?” (S2A10); “I totally believe academician Zhong Nanshan, who addressed the importance of TCM. I also realize the importance of quarantine, but we should try all the ways” (S3A3); “Many people blindly criticize TCM. I think such criticisms show disrespect to our traditional culture. Experts affirm TCM’s [effect], so I must buy SHL” (S2A9).

### 4.4. Conformity Behavior in the Purchase Craze of Shuanghuanglian

We find that conformity is another important variable for stimulating panic purchases. Referring to the existent literature, we define conformity as a modification of an individual’s previous behavior in the direction of a norm, stated or implied. The individual tendency of conformity behavior is higher during an emergency situation [65,66]. Conformity behavior can be explained by the social contagion model. Social behavior and decisions are “contagious”, too. The copying of behavior is attributed to two factors: the informational benefit (e.g., inferring hidden, private information others may know) and direct benefitial effects resulting from coordinated actions or social pressure [67].

Based on the present observed purchase craze, we summarized three sorts of conformity behavior. The first type was under the family’s pressure or neighbors’ influence. In this situation, an individual’s perception is either ambiguous or precise. Two interviewees showed conformity behavior with certain perceptions. Their attitudes to SHL were negative, but they still stood in the queue to respect the desires of their family members. For example: “Actually I highly doubt SHL’s effect, but I also have to follow my mom’s orders. She firmly believes in it” (S2A4); “Personally, I do not believe SHL. You can see that much information on social media and lots of people transmit. Elders have insufficient capacity to distinguish between information and they tend to believe it. I have no choice but to satisfy them” (S2A2).

The second type is blind conformity, which means that curiosity is the driver of purchase. In this situation, individuals have no actual perception, but the purchase process formed their initial perceptions. Six interviewees admitted that they were curious when they saw the queue in front of the pharmacy and joined the queue as a result of others’ persuasion. For example: “I just passed by this pharmacy and was attracted by the line. Many people told us that SHL may have an effect, so I wanted to have a try”; “I have no idea. Other people buy it, so I will buy it” (S3A4).

The third type is the conformity of rational choice. Individuals may have normal perceptions and the purchase behavior is motivated by the rational balance among potential medical effect, time cost, and money cost. Four interviewees said the purchase was better than doing nothing, because SHL could have a potential positive effect and there was little time or money cost associated with purchasing it. For example: “Quarantine is completely effective. But the media reported that SHL had a certain effect and my family also hopes to try it. I think going outside is okay. After all, everyone needs their temperature tested when they go out. So, it is no risk if I come to buy SHL” (S3A10); “Isolation at home is so boring. If SHL is potentially effective, why would I not buy it? SHL is a cheap medicine for us. You see me, I am wearing a mask. It is no problem if I use full protections when I buy the medicine” (S1A4).

## 5. Discussion

Rumors form via particular mechanisms. In this section, we discuss why news about SHL became a health rumor. In addition, based on the case study, targeted communication methods are given in order to manage rumors.

### 5.1. How an Ordinary News Report Becomes a Rumor: A Simplified Process Model

**Figure 1:**
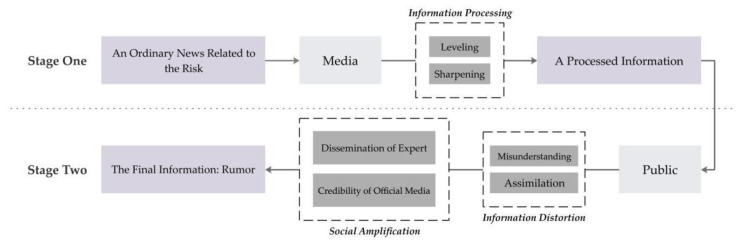
The forming mechanism of the Shuanghuanglian (SHL) rumor.

The purchase craze of SHL showed a process where an ordinary news report became a rumor. Following Allport and Postman’s framework, three factors can distort a piece of information and turn it into a rumor: leveling, sharpening, and assimilating. For leveling, a rumor tends to grow shorter and more concise, more easily grasped and told. Fewer words and fewer details are characteristics of a rumor. For sharpening, it is defined as the selective perception, retention, and reporting of a limited number of details from a broader context. Assimilation suggests that perception is, in no small extent, a question of psychology and attitude associated with habits, interests, and sentiments existing in the listener’s mind [31]. Based on this framework and our interview data, we established a simplified model to visualize the process of rumor formation (Figure 1).

From Figure 1, we divided the entire mechanism into two stages:

Stage one: This stage shows how media processes and distorts information. As mentioned, the media tends to retransmit complicated news into a brief and sketchy version. Such a process then levels and sharpens. From the media’s report, the media is prone to utilize attractive titles and sketchy content, which matches the most crucial issue that the public is concerned about and grasps the public’s attention. Although the media retransmits the whole content, an attractive title can be used, such as “Good news” and “Big news”. For instance, social media used a title inconsistent with the facts for attracting attention, such as “Big news! Tell the people around you ASAP: Here comes the medicine that can cure COVID-19” [68]. Therefore, people perceived SHL as being capable of remedying the novel coronavirus.

Stage two: This stage illustrates how a piece of processed information finally becomes a rumor. There are two key variables responsible for the variation of information and forming the final version of the rumor. The information distortion in the model represents people’s subjective attitudes and inaccurate decoding of the information. Two concepts of misunderstanding and assimilation are used to generalize the cause of information distortion, showing that people tend to systematically favor information that is consistent with their beliefs and preferences [69]. According to our interviews, ordinary people failed to understand medical jargon and professional terms, and were prone to regarding “inhibition” as a synonym of “prevention” and “remedy”, as well as being prone to making decisions based on this perception. In addition, our interviews proved that people’s belief in TCM aroused keen interest in purchasing SHL. In addition to information distortion, expert and credible sources who allegedly supported the purchase of SHL jointly caused social amplification, intensifying people’s perception and social response, and further influencing people’s behavior. During a high-risk event, trust in experts and official media is mediated between risk perception and risk behavior because people rely on credible information to make decisions [52]. Social amplification enhances the stability of a rumor and broadens its extension by reinforcing people’s health behavior.

### 5.2. Effective Health Communication: Implications for Managing Health Rumors during a Public Health Emergency

Based on this purchase craze, we suggest some implications for managing rumors following principles of effective health communication.

Communication is a central human process that enables individual and collective adaptation to health risks at many different levels [70]. During a public health emergency, the priority of health communication is to adjust people’s cognition by using simple, intact, and factual expression to deliver complex uncertainty. Generally, people know the health risks they encounter, but people’s cognition only rests on the surface instead of on the scientific rationales behind the risk. People assemble fragmentary beliefs of the risk into a “mental model”, which they then use to reach conclusions [61,71]. In this case, the inaccurate cognition in medicine effect resulted in the rumor’s occurrence. Therefore, health communication ought to disseminate health and risk information via simple expressions, which people can understand easily, rather than putting forward the medical terminology directly. Therefore, perceptions of the public, particularly when linked to media representations, are highly dependent on how messages are framed [72].

Effective health communication during a public health emergency is a pre-informed procedure with credible, professional, and authoritative information sources. Experts ought to play a vital role in communication instead of the media. What a health communication should contain depends on what audience members intend to do with it. In other words, people always need advice about what they should do rather than a post-explanation. In the SHL case, after the purchase craze, experts were asked to refute the rumor and explain the real meaning of the information [73]. If communication fails to deliver critical and accurate information in advance, it may leave information receivers with an illusion that what they hear is reality, which causes a chain reaction of worsening impacts and even impairs the credibility of information releasing. Effective risk communications require authoritative and trustworthy sources, and experts have an undisputed role in communicating risk information [71,74]. Existing studies have argued that the news media and the Internet have been criticized as they often publish inaccurate, sensationalized, or misleading stories that are not necessarily the most scientifically significant ones. This is because the news is not peer-reviewed and many health reporters have no health or science training [72]. During the COVID-19 outbreak in China, some experts earned a high reputation due to their timely and effective communication with public, like Chinese famous doctors Zhong Nanshan and Zhang Wenhong, both of whom have become credible sources for informing the public about the outbreak [48]. The objective of effective health communication is to lead people’s proactive behavior of prevention instead of passive obedience. Dr. Zhong has very high reputation among Chinese people because his outstanding performance in SARS outbreak. Therefore, Dr. Zhong is a credible source of health communication. Dr. Zhang Wenhong, an expert responsible for communication on behalf of the authorities, said that combating the outbreak depends on people’s collaboration, which is associated with clearly and plainly communicating with the public [75].

Following the above analysis, three principles can help conduct effective health communication and health rumor management:

♦ ***Maintaining the intelligibility of information:*** There should be unitized alternatives of medical terms to inform the public, such as plain language and vocabularies. Due to the different health literacy of people, it is appropriate to use simple and straight language for health communication. When communicating public health information, equivocal expressions are most effective for avoiding overly certain predictions [76]. If popular interpretations of reality are understood, then health communicators are more capable of translating technical and scientific concepts into understandable messages [72]. In addition, health communication in a public emergency should assign priority to different information. Therefore, communicators need to find out what the public needs and wants, and to make their information more interesting, relevant, and attractive to the target audience [77].
♦ ***Keeping the accuracy of information:*** Factual information is a key factor for preventative rumor management. Thus, never level and sharpen a piece of information into a sentence that may mislead people [78]. According to our analysis, SHL rumors relied on people’s misunderstanding that “SHL oral liquid can inhibit the novel coronavirus”. Dissemination of risk information should be intact and evidence-based, rather than garbling a statement for highlighting achievements. The media ought to avoid utilizing exaggerated titles—such as “big news” and “breaking news”—with incomplete information, which may mislead people’s perceptions and behaviors. The aim of health communication in an emergency is to narrow uncertainty, explaining and understanding patient concerns, even when they cannot be resolved. This results in a significant drop in anxiety [70].
♦ ***Enhancing the credibility of information:*** Expert dissemination ought to parallel with media reports when the contents are linked to professional issues, such as epidemiological investigation and advances in medicine research, by direct and plain expressions. In order to correct people’s health perceptions and behaviors, experts should suggest the strength of the recommendation and the quality of the evidence that supports the recommendation, which refers to the practice of the National Institutes of Health, so that they can offer a numerical and hierarchical cognition about probability and effectiveness and help people make a scientific judgment and rational decision [79]. Additionally, in view of extensive social media usage, experts can convey accurate health information by using social media in order to broaden the scope of information dissemination. For instance, famous expert Zhang Wenhong has used Weibo to deliver health information about COVID-19. Dr. Zhang’s Weibo attracted over 3 million followers within one month [80].

**Figure 2:**
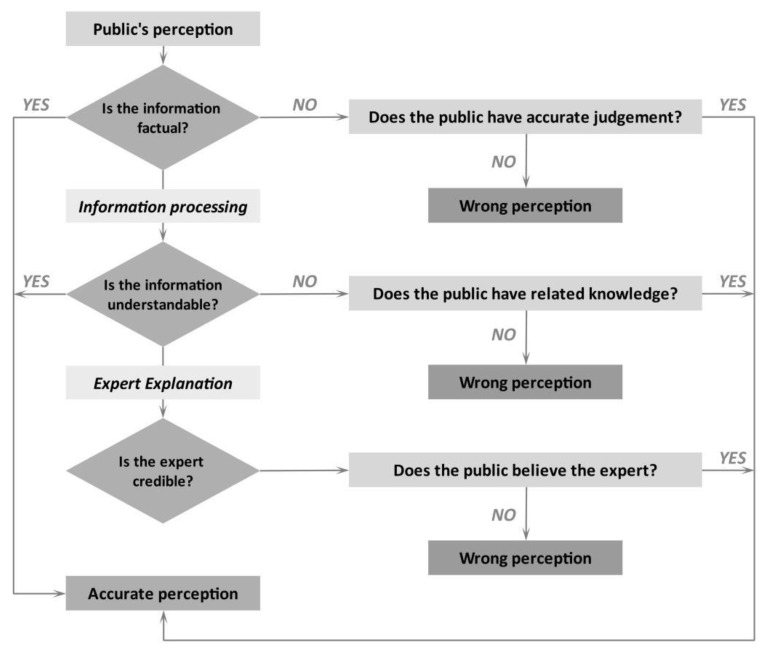
Tree diagram of public’s perception on health information.

Integrating our research findings and three principles of health communication, we propose a simplified tree diagram to illustrate how health communications influence the public’s perception on health information (Figure 2).

## 6. Conclusions

Rumors always occur during public health emergencies and often mislead people’s health perceptions and behaviors. Taken together, this article details what factors influence people’s perceptions and behaviors regarding health rumors, specifically regarding the SHL purchase craze during the COVID-19 outbreak in China. Following theoretical and empirical analysis, we argued that expertise in a medical issue, psychology for dealing with uncertainty, social and cultural factors, and conformity jointly reinforces people’s belief in rumors. In addition, official media and experts’ suggestions amplify people’s perception.

For managing health rumors during a public health emergency, we firstly described how a piece of information becomes a rumor via leveling, sharpening, and assimilation. Furthermore, we indicated that an effective health communication for rumor management depends on prior prevention and thus recommended three main strategies of health communication: withholding information intelligibility, ensuring information accuracy, and enhancing information credibility. These three strategies aim to lead people to establish accurate health perceptions and perform scientific health behaviors step-by-step, thereby enhancing the effectiveness of risk management. The specialty of this case is that cultural factors profoundly impact people’s perceptions and behavior. Therefore, health communication should use the advantage of cultural factor to enhance people’s accurate perception. Moreover, this case shows that the perception divergence amongst different people may mislead or change people’s health behavior due to specific pressures from acquaintances or families. Thus, an individual ought to be responsible for correcting other people’s perception at the level of family and community.

Our findings are not without limitations. In this article, we utilized too few samples to analyze the whole process of a health rumor, which also reflects the explanatory power of cases or interview data. In addition, managing health rumors by using effective health communication is a practical issue with implications in the real world. Therefore, our recommendations for health rumor management communication should be tested through related empirical research in the future. For future research, we will consider if demographic characters will influence people’s perception and health behavior, and comparative study should be conducted based on the same phenomenon in different countries.

## Figures and Tables

**Table 1:** The population demographics of interviewees.

Site							
	**Valid Interviewees**				**Actual Interviewees**		
Shenzhen	10				12		
Shanghai	10				13		
Yingkou	10				15		
**Age range**							
Under 18	18–30		31–60			Over 60	
3	10		12			5	
**Gender**							
Male			Female				
14			16				
**Income**							
	High income			Medium income	Low income		Secretive
Occupation	Governmental official	Corporate manager		Civil servant/ corporate employee	Worker/ peasant/ student		
	2	1		6	18		3

**Source:** Interviewees’ personal information. **Note:** The valid interviewee excludes the interviewees who declined the interview.
